# Role of serum ceruloplasmin in the diagnosis of Wilson's disease: A large Chinese study

**DOI:** 10.3389/fneur.2022.1058642

**Published:** 2022-12-07

**Authors:** Yue Yang, Wenjie Hao, Taohua Wei, LuLu Tang, Nannan Qian, Yulong Yang, Hu Xi, Shijie Zhang, Wenming Yang

**Affiliations:** ^1^Department of Graduate, Anhui University of Chinese Medicine, Hefei, Anhui, China; ^2^Department of Neurology, The First Affiliated Hospital of Anhui University of Chinese Medicine, Hefei, Anhui, China; ^3^Xin'an Medical Education Ministry Key Laboratory, Hefei, Anhui, China

**Keywords:** Wilson's disease, Chinese cohort, serum ceruloplasmin, diagnosis, phenotype, genotype

## Abstract

**Background:**

Conventionally, serum ceruloplasmin levels below the lower reference limit (0. 20 g/L) is considered a diagnostic cutoff point for Wilson's disease (WD). However, the lower reference limit varies with assay methodologies and the individuals in the included studies. The objective of this study was to determine the optimal cutoff value of serum ceruloplasmin levels for the diagnosis of WD in a large Chinese cohort and to identify factors associated with serum ceruloplasmin.

**Methods:**

The cutoff value of ceruloplasmin levels was developed based on a retrospective derivation cohort of 3,548 subjects (1,278 patients with WD and 2,270 controls) and was validated in a separate validation cohort of 313 subjects (203 patients with WD and 110 controls). The performance of immunoassay was tested by receiver operating characteristic curve (ROC) analysis, and differences among the groups were analyzed by using the Mann–Whitney *U*-test and the Kruskal–Wallis test.

**Results:**

The conventional cutoff of serum ceruloplasmin levels of <0.2 g/L had an accuracy of 81.9%, which led to a false-positive rate of 30.5%. The optimal cutoff of the serum ceruloplasmin level for separating patients with WD from other participants was 0.13 g/L, as determined by ROC analysis. This cutoff value had the highest AUC value (0.99), a sensitivity of 97.0%, and a specificity of 96.1%. Moreover, it prevented unnecessary further investigations and treatments for 492 false-positive patients. By determining the correlation between serum ceruloplasmin and phenotypes/genotypes in patients with WD, we found that the serum ceruloplasmin level was lower in early-onset patients and higher in late-onset patients. Interestingly, patients with the R778L/R919G genotype had higher serum ceruloplasmin levels than patients with other hot spot mutation combinations.

**Conclusion:**

Our work determined the optimal cutoff value of serum ceruloplasmin levels for the diagnosis of WD and identified differences in serum ceruloplasmin levels with respect to the age of symptom onset and *ATP7B* mutations, which may provide some valuable insights into the diagnosis and counsel of patients with WD.

## Introduction

Wilson's disease (WD) is an autosomal recessive disorder of copper accumulation caused by mutations in the *ATP7B* gene (1). Copper gets deposited in different organs, and the spectrum of clinical manifestations varies from progressive hepatic damage and neurological deterioration to atypical cardiac and osteoarticular involvement (2). Due to variable clinical presentations, it is difficult to accurately and timely identify WD. In 2003, Ferenci et al. (3) proposed a scoring system for the diagnosis of WD (Leipzig score) that was based on a combination of clinical manifestations, laboratory tests, and genetic analysis. Liver histology, as an invasive method, is uncommon for the routine diagnosis of WD; furthermore, it has poor sensitivity at an early stage (4). Genetic analysis can allow unequivocal diagnosis if disease-causing mutations on both chromosomes are identified. However, it is a cumbersome and costly test. Moreover, the significance of many mutations that occur in the *ATP7B* gene is undetermined (5). In addition, Kayser–Fleischer (K–F) rings are a typical ophthalmologic symptom found in patients with WD. These rings are common in patients with neurological WD, but they are less common in hepatic and asymptomatic subtypes (6).

In general, the serum ceruloplasmin test is the most widely accessible biochemical test for WD. In 1952, Scheinberg and Gitlin first reported that serum ceruloplasmin is deficient in patients with WD (7). Further studies demonstrated that low serum ceruloplasmin levels provided strong evidence for the diagnosis of WD, so the measurement of serum ceruloplasmin levels was recommended as the first step in screening for WD (8, 9). Conventionally, serum ceruloplasmin levels below the low reference limit (0.20 g/L) is considered a diagnostic cutoff point for WD (1). However, serum ceruloplasmin levels are also found reduced in cases of copper deficiency, chronic liver disease, protein-losing enteropathy, and hereditary aceruloplasminemia (10). Therefore, the use of low reference limit for serum ceruloplasmin may lead to unacceptably high numbers of false-positives, which may trigger further investigations and overtreatments. Mak et al. (11) recommended that each laboratory should establish a cutoff value of serum ceruloplasmin levels for the diagnosis of WD according to the local population, rather than using the lower reference limit.

The objective of this study was to determine the optimal cutoff value of serum ceruloplasmin levels for the diagnosis of WD in a large Chinese cohort and identify factors associated with serum ceruloplasmin.

## Patients and methods

### Patients

All patients who underwent a serum ceruloplasmin test from 1 January 2016 to 2 September 2019 in the First Affiliated Hospital of Anhui University of Chinese Medicine were eligible for this study. We divided the derivation and validation cohorts based on the time point of 30 August 2016. In all the screened patients, the diagnosis of WD was based on a Leipzig score of 4 or more (3). Appropriate controls include either heterozygous subjects or those with various diseases other than WD. In addition, healthy controls with no clinical evidence of WD were recruited for our study. The Ethics Committee of The First Affiliated Hospital of Anhui University of Chinese Medicine approved this study.

### Laboratory methods

Serum ceruloplasmin concentrations were measured by immuno-nephelometric assays with anti-serum to human ceruloplasmin on the IMMAGE Immunochemistry System (Beckman Coulter, USA). Dilution linearity was observed in the range of 0~0.800 g/L (with a regression coefficient of *r*^2^ > 0.990). The exonic sequences and the intron–exon boundaries of *ATP7B* were amplified using a polymerase chain reaction, as previously described (12).

### Statistical analysis

All statistical analyses were performed by using SPSS version 23 (IBM, Armonk, NY). Quantitative data are expressed as mean ± SD or median and interquartile range (IQR) based on normality or nonnormality. A receiver operating characteristic curve (ROC) was generated to determine the most appropriate cutoff value of serum ceruloplasmin levels for the diagnosis of WD. The statistical analysis of differences between the two groups was performed by using the Mann–Whitney *U*-test. The Kruskal–Wallis test and the *post hoc* test were used to compare differences among three or more groups. A *P*-value <0.05 was considered statistically significant.

## Results

### Characteristics of patient cohorts

As previously reported (12), a definitive diagnosis of WD was confirmed in 1,302 index patients based on the Leipzig score at The First Affiliated Hospital of Anhui University of Chinese Medicine from 31 August 2016 to 2 September 2019. Of these 1,302 patients, 1,278 patients with WD were given ceruloplasmin tests (for demographics, see Table 1). The patients with WD were compared with three other cohorts. (1) The reference interval of serum ceruloplasmin levels was obtained from healthy subjects who were 18–69 years old; of the 418 healthy subjects, 213 were male. (2) A total of 1,772 patients with various diseases other than WD were recruited, including 1,118 patients with hepatic dysfunction, 414 patients with neurological deficits, and 240 patients with other diseases. Our study included 1,051 male non-WD patients, with a median age of 28 (IQR 18~42) years. (3) In addition, 80 heterozygous relatives of patients with WD were enrolled. Of the 80 heterozygous relatives, 52 (64.2%) were male, with a median age of 22 (IQR 12~32) years.

**Table 1:** Demographic characteristics of patients with WD.

**Parameter**			**Case (*n*)**
Male/Female			764/514
Age at manifestation	Range	1.75~64.75	1,278
(years)	Mean ± SD	17.00 ± 9.33	
Serum ceruloplasmin	Range	0.001~0.238	1,278
(g/L)	Median	0.029	
	Quantile (0.25~0.75)	0.016~0.052	
Kayser-Fleischer ring	Negative		180
	Positive		1,098
Onset form	Hepatic		307
	Neurologic		728
	Asymptomatic		218
	Others		25
*ATP7B* mutation	Homozygotes		201
	Heterozygotes		946
	One variant identified		112
	No variant identified		19

Moreover, 203 patients with WD and 110 controls (72 patients with hepatic dysfunction, 22 patients with neurological deficits, 11 patients with other diseases, and five heterozygous relatives of patients with WD) from 1 January 2016 to 30 August 2016 in our hospital were included, which constituted the validation cohort. The composition of the controls in the derivation cohort and validation cohort are provided in Supplementary Tables 1, 2.

### The diagnostic performance of serum ceruloplasmin

**Figure 1:**
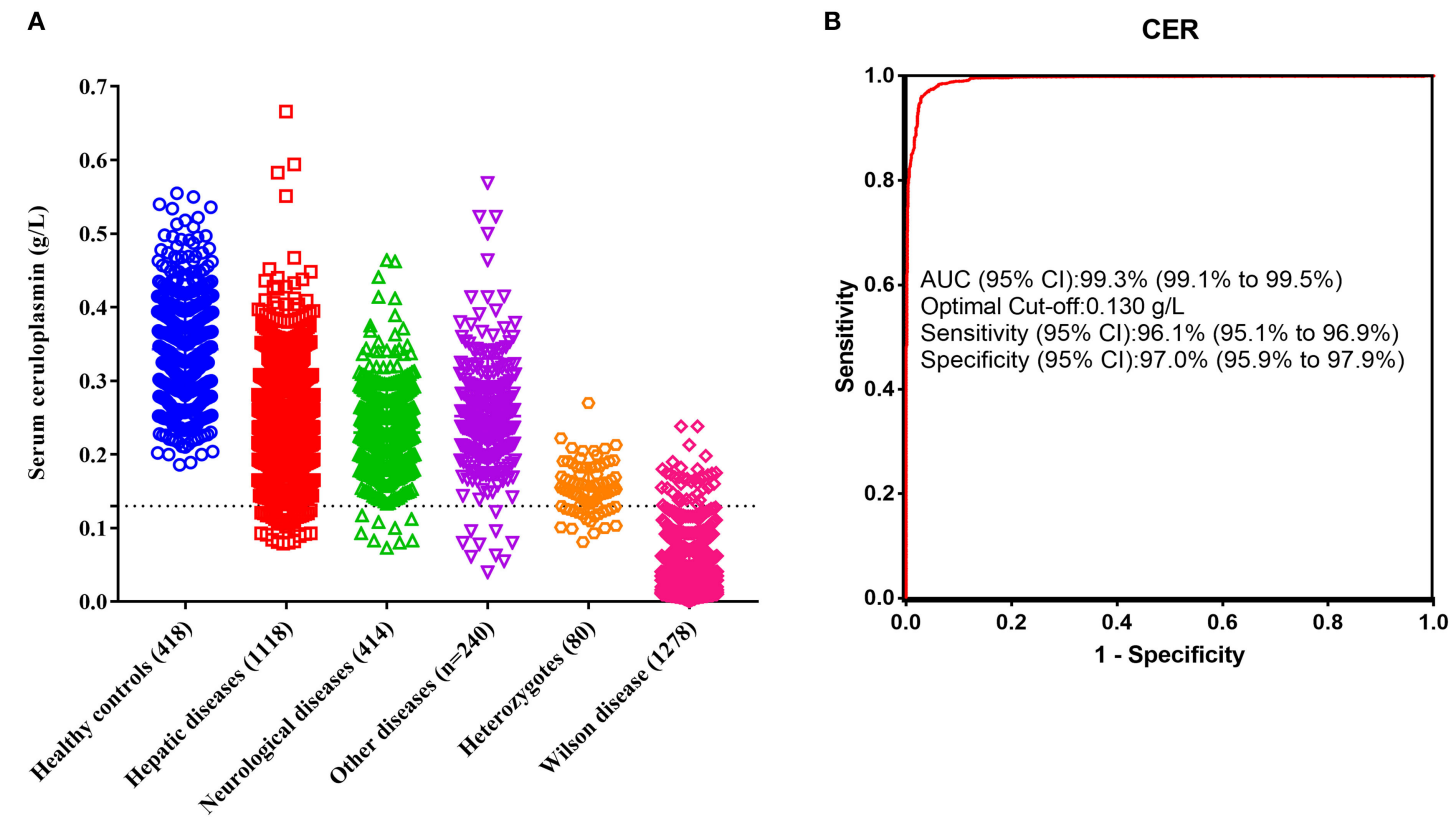
Diagnostic performance of serum ceruloplasmin. **(A)** Comparison of serum ceruloplasmin concentrations among healthy controls, patients with hepatic diseases, patients with neurological diseases, patients with other diseases, heterozygotes, and patients with WD. **(B)** ROC of serum ceruloplasmin concentration for the diagnosis of WD.

The range of serum ceruloplasmin concentrations for the reference interval group was 0.186~0.555 g/L, and only two of these individuals had concentrations <0.20 g/L. Serum ceruloplasmin levels were significantly lower in the patients with WD (0.029 g/L, IQR 0.016~0.052) than in the heterozygous relatives (0.153 g/L, IQR 0.130~0.182) and non-WD patients with liver dysfunction (0.242 g/L, IQR 0.196~0.288), neurological deficits (0.225 g/L, IQR 0.188~0.266), and other diseases (0.246 g/L, IQR 0.206~0.292; Figure 1A).

The conventional ceruloplasmin cutoff of <0.20 g/L was used, and WD was diagnosed in 1,275 patients, with a sensitivity of 99.8%, a specificity of 69.5%, and an accuracy of 81.9%. However, 564 false-positive cases were noted in this study, resulting in a 30.5% false-positive rate. The most common etiologies of false positivity in the present study were non-WD hepatitis, liver failure, nephrotic syndrome, and heterozygous relatives. Of the 1,118 patients with liver dysfunction, 27.10% had ceruloplasmin levels <0.20 g/L. Particularly, those with liver failure had lower serum ceruloplasmin levels (0.168 g/L, IQR 0.141~0.236), and 66.40% (83/125) of the patients had ceruloplasmin levels <0.20 g/L. These results confirm that a ceruloplasmin level <0.20 g/L is unsuitable as the optimal cutoff value for the diagnosis of WD.

Therefore, we performed ROC analysis using data from 1,278 patients with WD, 80 heterozygous relatives, and 1,772 non-WD patients. The optimal cutoff of serum ceruloplasmin levels to separate WD from other participants determined by ROC analysis was 0.13 g/L, and the area under the curve was 99.3% (Figure 1B). The optimal cutoff of 0.13 g/L resulted in a sensitivity of 97.0%, a specificity of 96.1%, and an accuracy of 96.5%. When a cutoff value of 0.13 g/L was used in the validation cohort, 197 patients with WD and 105 controls were correctly identified, with a sensitivity of 97.0%, a specificity of 95.5%, and an accuracy of 96.5%. Overall, the optimal cutoff of 0.13 g/L provided both higher specificity and accuracy than the conventional cutoff level of 0.20 g/L.

### Correlation between serum ceruloplasmin and phenotypes

**Figure 2:**
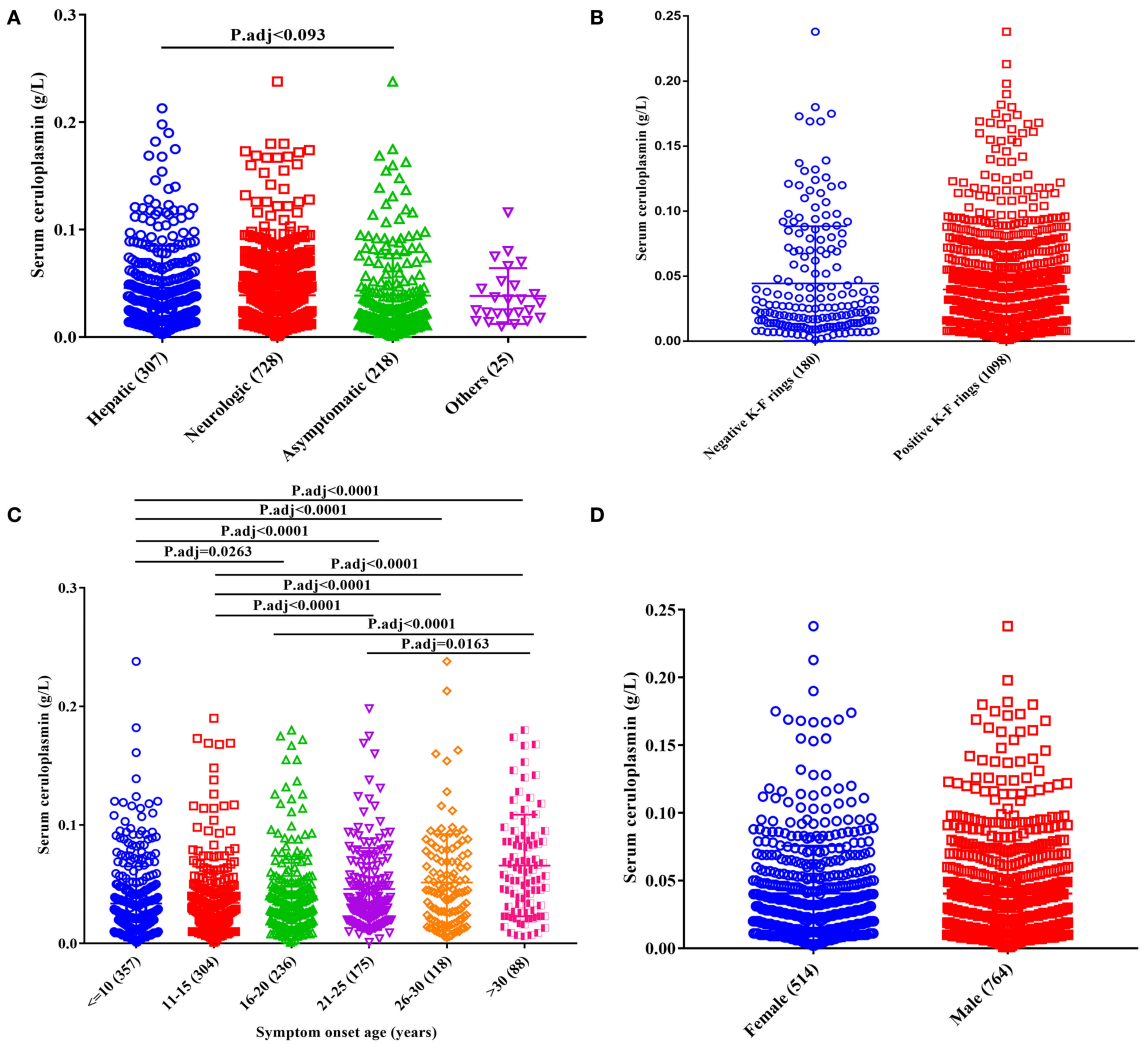
Effect of clinical phenotypes on serum ceruloplasmin levels of patients with WD. **(A)** Clinical subtypes; **(B)** presence of K–F rings; **(C)** age at onset; **(D)** sex.

The serum ceruloplasmin levels were significantly lower in the asymptomatic subtype (0.024 g/L, IQR 0.014~0.047) than in the hepatic subtype (0.034 g/L, IQR 0.016~0.063), and the comparisons among other groups were not statistically significant (Figure 2A).

With respect to the age of symptom onset, the serum ceruloplasmin level was lower in patients with an age of symptom onset <10 years (0.022 g/L, IQR 0.013~0.043) and higher in patients with an age of symptom onset >30 years (0.059 g/L, IQR 0.029~0.088; Figure 2C).

Furthermore, no significant differences in ceruloplasmin levels were found based on sex (female vs. male; Figure 2D) or the presence of a K–F ring (Figure 2B).

### Correlation between serum ceruloplasmin and genotypes

**Figure 3:**
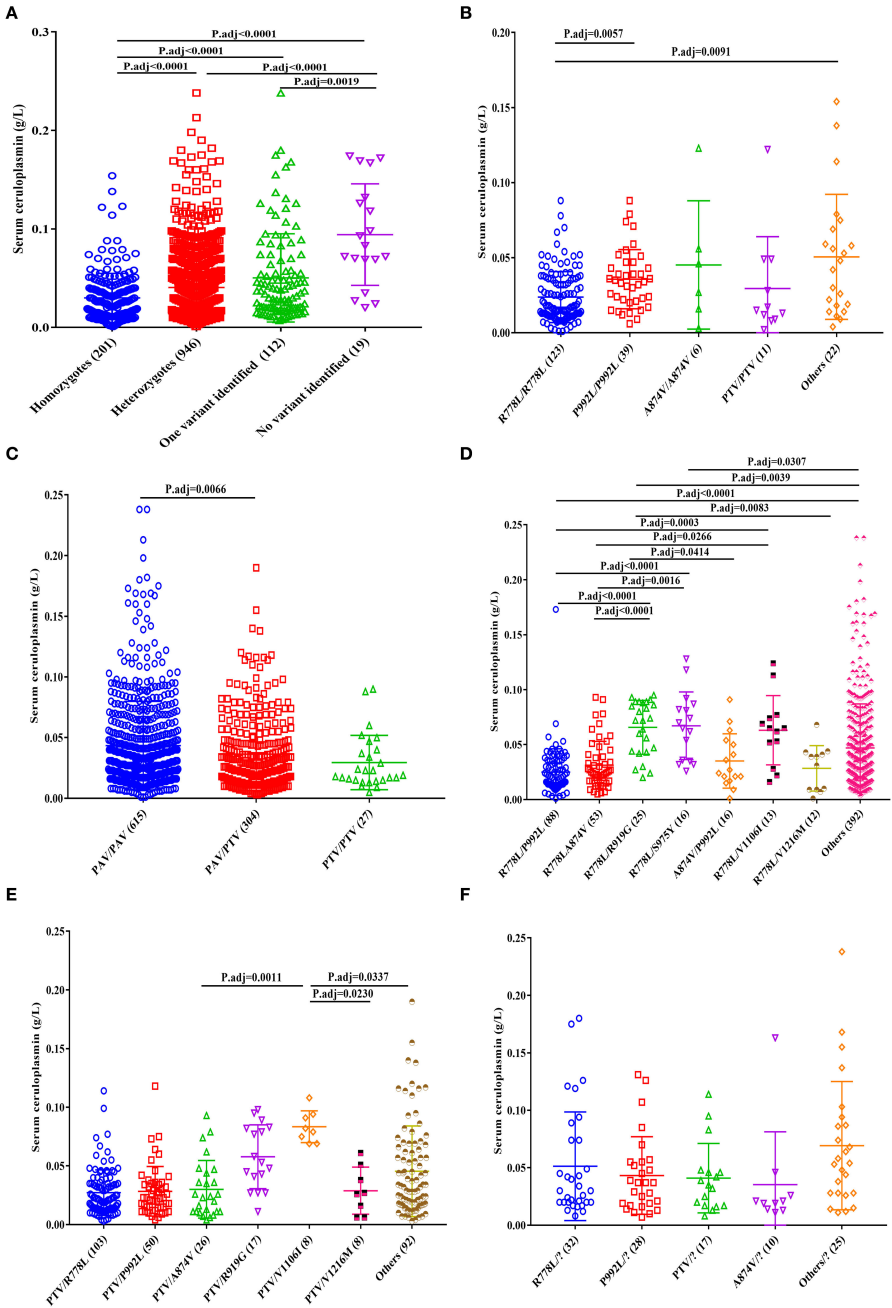
Effect of common *ATP7B* genotypes on serum ceruloplasmin levels of patients with WD. **(A)** Total, **(B)** homozygotes, **(C)** heterozygotes, **(D)** PAV/PAV, **(E)** PTV/PAV, and **(F)** one variant identified.

According to previous sequencing results (12), 1,147 patients harbored two disease-causing variants (201 homozygotes and 946 compound heterozygotes). Moreover, 112 WD cases had only one variant with no second mutation detected, and 19 patients with no potential disease-causing variants were identified. Among the four genotypes, homozygous patients had the lowest serum ceruloplasmin levels (0.024 g/L, IQR 0.013~0.040), and patients with no variant identified had the highest serum ceruloplasmin levels (1.036 g/L, IQR 1.019~1.067; Figure 3A).

For the homozygous patients, the patients with R778L/R778L (0.017 g/L, IQR 0.011~0.036) had lower serum ceruloplasmin levels than those with P992L/P992L (0.034 g/L, IQR 0.021~0.047) and other homozygous genotypes (0.045 g/L, IQR 0.017~0.071; Figure 3B).

Among the heterozygous patients, the patients with PAV/PAV (0.032 g/L, IQR 0.018~0.057) had higher serum ceruloplasmin levels than those with PTV/PAV (0.027 g/L, IQR 0.014~0.047; Figure 3C). In the patients with PAV/PAV, the patients with R778L/P992L (0.020 g/L, IQR 0.013~0.035) had lower serum ceruloplasmin levels, and the patients with R778L/R919G (0.072 g/L, IQR 0.044~0.089) and R778L/S975Y (0.068 g/L, IQR 0.035~0.086) had higher serum ceruloplasmin levels (Figure 3D). In the patients with PTV/PAV, the patients with PTV/V1106I (0.081 g/L, IQR 0.071~0.093) had higher serum ceruloplasmin levels than those with PTV/A874V (0.024 g/L, IQR 0.011~0.045), PTV/V1216M (0.027 g/L, IQR 0.009~0.048), and other PTV/PAV genotypes (0.033 g/L, IQR 0.017~0.066; Figure 3E).

In addition, there were no significant differences in the ceruloplasmin levels among patients with one variant (Figure 3F).

## Discussion

In general, the reference range of serum ceruloplasmin concentrations by immunoassay in healthy adults is between 0.20 and 0.40 g/L. Conventionally, ceruloplasmin levels below the low reference limit (0.20 g/L) are considered a diagnostic cutoff for WD (1). However, the lower reference limit varies with assay methodologies and individuals in the included studies (with differences in age, ethnicity, and clinical presentations) (11). Therefore, some studies have attempted to determine the cutoff value of serum ceruloplasmin levels for the diagnosis of WD based on the local population. Through a literature review, we found that five studies evaluated the thresholds of ceruloplasmin, which ranged from 0.14 to 0.19 g/L. However, these studies were mostly from Europe and used a small number of patients with WD (11, 13–16). Recently, a systematic review of the use of the ceruloplasmin test for the diagnosis of WD indicated key biases in participant selection and reference standards, which indicate that the cutoff used for ceruloplasmin requires external validation (17). Considering the differences in assay methodology and individual selection, further studies with a larger number of patients are necessary.

Thus, we recruited a large Chinese cohort to determine the diagnostic accuracy of serum ceruloplasmin for WD diagnosis, which included 1,278 patients with WD from 30 different provinces of China. Moreover, 1,772 patients with various diseases other than WD were recruited, including 1,118 patients with hepatic dysfunction, 414 patients with neurological deficits, and 240 patients with other diseases. In addition, 80 heterozygous subjects and 418 healthy controls were enrolled. Our study showed that the conventional cutoff value of serum ceruloplasmin levels of <0.2 g/L had a sensitivity of 99.8%, a specificity of 69.5%, and a false-positive rate of 30.5%. The optimal cutoff value of serum ceruloplasmin levels for the separation of patients with WD from other participants was 0.13 g/L, as determined by the ROC analysis. When the threshold of 0.13 g/L was used, sensitivity decreased by only 2.8%, but specificity increased by 26.6%. Moreover, it prevented 492 false-positive patients from undergoing unnecessary further investigations and treatments.

Notably, the cutoffs used for ceruloplasmin levels for diagnosing WD are method-dependent. The serum ceruloplasmin level can be measured enzymatically or immunologically. However, the measurements of serum ceruloplasmin oxidase activity are unavailable in routine laboratory tests, and serum ceruloplasmin is commonly measured by performing immunoassays. Discrepant measurement results were also found for ceruloplasmin levels when measured using different immunoassay methods, which may vary by ±20% (18). Ceruloplasmin is an acute-phase reactive protein, which is often confounded by many other diseases, including decompensated liver failure, nephrotic syndrome, protein-losing enteropathy, and acquired copper deficiency. In WD patients with acute liver failure, serum ceruloplasmin levels <0.2 g/L by nephelometry provided a diagnostic sensitivity of 56% and specificity of 63% (19). Therefore, a single-test strategy to diagnose or exclude WD is irrational.

In addition, some studies have sought to evaluate whether serum ceruloplasmin levels are associated with a particular phenotype/genotype of the disease. Cheng et al. (20) discovered that higher serum ceruloplasmin levels indicated a greater possibility of the hepatic subtype, while lower serum ceruloplasmin levels were associated with the neurological subtype. Dong et al. (6) indicated that serum ceruloplasmin levels differed among asymptomatic patients and osteomuscular or neurological deficit patients with WD. Moreover, no significant difference was detected in ceruloplasmin levels in WD patients with or without K–F rings (6). In addition, some studies observed lower serum ceruloplasmin levels in patients with R778L homozygous mutations than in patients without R778L mutations (21). Another study reported that low serum ceruloplasmin levels were associated with truncating mutations in the *ATP7B* gene (22).

Therefore, as the second part of our study, we identified factors associated with serum ceruloplasmin. In terms of clinical phenotype, serum ceruloplasmin levels were significantly higher in the hepatic subtype than in the asymptomatic subtype. With respect to the age of symptom onset, the serum ceruloplasmin level was lower in early-onset patients and higher in late-onset patients. Analysis of the relationships among the genotypic factors revealed that the homozygous patients had lower serum ceruloplasmin levels than heterozygous patients or patients with one or no variant. Among the heterozygous patients, the patients with PAV/PAV had higher serum ceruloplasmin levels than those with PTV/PAV. Interestingly, patients with the R778L/R919G genotype had higher serum ceruloplasmin levels than patients with other common variants (PTV, R778L, P992L, A874V, and R919G; Supplementary Figure 1).

In summary, our work determined the optimal cutoff value of serum ceruloplasmin levels for the diagnosis of WD and identified differences in serum ceruloplasmin levels with respect to the age of symptom onset and ATP7B mutations, which may provide some valuable insights into the diagnosis and counsel of patients with WD.

## Data availability statement

The data presented in the study are available on request from the corresponding authors.

## Ethics statement

The studies involving human participants were reviewed and approved by the Ethics Committee of the First Affiliated Hospital of Anhui University of Chinese Medicine. Written informed consent to participate in this study was provided by the participants' legal guardian/next of kin.

## Author contributions

WY, SZ, and YueY designed the study. YueY performed data analysis and wrote the manuscript. WH, TW, LT, YulY, NQ, and HX were responsible for sample collection and identifying clinical information. WY and SZ revised the manuscript. All authors reviewed and agreed to publication of the final version of the manuscript.

## Supplementary material

Supplementary Table 1 Composition of controls group in derivation cohort and their serum ceruloplasmin levels.

Supplementary Table 2 Composition of controls group in validation cohort and their serum ceruloplasmin levels.
