# Supplementary material for: Role of serum ceruloplasmin in the diagnosis of Wilson's disease: A large Chinese study
Source: Front Neurol. 2022 Dec 7;13:1058642. doi: 10.3389/fneur.2022.1058642 (PMC9768184; doi:10.3389/fneur.2022.1058642)
Supplement: Supplementary Table 1 — Composition of controls group in derivation cohort and their serum ceruloplasmin levels. [file Table_1.doc]

| **Classification (n)** | **Composition (n)** | **Serum ceruloplasmin (g/L)**  **Quantile (0.25~0.75)** |
| --- | --- | --- |
| Hepatic dysfunction  (n=1118) | Chronic hepatitis (n=307) | 0.207~0.304 |
|  | Cirrhosis (n=243) | 0.169~0.266 |
|  | Acute hepatitis (n=156) | 0.231~0.306 |
|  | Fatty liver disease (n=111) | 0.186~0.268 |
|  | Acute liver failure (n=75) | 0.143~0.212 |
|  | Autoimmune hepatitis (n=62) | 0.252~0.369 |
|  | Chronic liver failure (n=50) | 0.133~0.236 |
|  | Drug-induced liver injury (n=49) | 0.181~0.261 |
|  | Other liver disease (n=65) | 0.246~0.265 |
| Neurological deficits  (n=414) | Parkinsonism (n=89) | 0.188~0.269 |
|  | Essential tremor (n=49) | 0.216~0.288 |
|  | Transient ischemic attack (n=31) | 0.179~0.288 |
|  | Cerebral palsy (n=30) | 0.179~0.250 |
|  | Ataxia (n=29) | 0.188~0.271 |
|  | Mental disorder (n=28) | 0.155~0.246 |
|  | Epilepsy (n=25) | 0.181~0.257 |
|  | Huntington disease (n=19) | 0.184~0.264 |
|  | Dystonia (n=17) | 0.184~0.240 |
|  | Mutiple tics-coprolalia syndrome (n=14) | 0.258~0.294 |
|  | Peripheral neuropathy (n=11) | 0.200~0.276 |
|  | Multiple system atrophy (n=6) | 0.185~0.252 |
|  | Other neurological disorders (n=66) | 0.191~0.263 |
| Other diseases (n=240) | Diabetes mellitus (n=58) | 0.196~0.324 |
|  | Rheumatic diseases (n=32) | 0.195~0.283 |
|  | Nephrotic syndrome (n=11) | 0.061~0.201 |
|  | Gastropathy (n=11) | 0.178~0.209 |
|  | Other systemic diseases (n=128) | 0.227~0.294 |
| Heterozygous (n=80) |  | 0.130~0.182 |
| Healthy controls (n=418) |  | 0.286~0.399 |

**Supplementary Table1. Composition of controls group in derivation cohort and their serum ceruloplasmin levels.**
