# Supplementary material for: Role of serum ceruloplasmin in the diagnosis of Wilson's disease: A large Chinese study
Source: Front Neurol. 2022 Dec 7;13:1058642. doi: 10.3389/fneur.2022.1058642 (PMC9768184; doi:10.3389/fneur.2022.1058642)
Supplement: Supplementary Table 2 — Composition of controls group in validation cohort and their serum ceruloplasmin levels. [file Table_2.doc]

| **Classification (n)** | **Composition (n)** | **Serum ceruloplasmin (g/L)**  **Quantile (0.25~0.75)** |
| --- | --- | --- |
| Hepatic dysfunction  (n=72) | Fatty liver disease (n=16) | 0.195~0.247 |
|  | Chronic hepatitis (n=14) | 0.183~0.233 |
|  | Cirrhosis (n=8) | 0.168~0.394 |
|  | Acute hepatitis (n=8) | 0.244~0.313 |
|  | Chronic hepatitis (n=8) | 0.140~0.342 |
|  | Drug-induced liver injury (n=6) | 0.182~0.260 |
|  | Autoimmune hepatitis (n=7) | 0.247~0.320 |
|  | Other liver disease (n=5) | 0.172~0.218 |
| Neurological deficits  (n=22) | Parkinsonism (n=7) | 0.181~0.273 |
|  | Transient ischemic attack (n=2) | 0.209~0.237 |
|  | Cerebral palsy (n=2) | 0.166~0.194 |
|  | Ataxia (n=2) | 0.200~0.230 |
|  | Huntington disease (n=2) | 0.216~0.225 |
|  | Dystonia (n=2) | 0.246~0.273 |
|  | Other neurological disorders (n=5) | 0.149~0.199 |
| Other diseases (n=11) | Rheumatic diseases (n=4) | 0.161~0.307 |
|  | Diabetes mellitus (n=2) | 0.323~0.344 |
|  | Nephrotic syndrome (n=3) | 0.078~0.198 |
|  | Other systemic diseases (n=2) | 0.152~0.175 |
| Heterozygous (n=5) |  | 0.090~0.156 |

**Supplementary Table2. Composition of controls group in validation cohort and their serum ceruloplasmin levels.**
