# Supplementary figures and images for: Role of serum ceruloplasmin in the diagnosis of Wilson's disease: A large Chinese study
Source: Front Neurol. 2022 Dec 7;13:1058642. doi: 10.3389/fneur.2022.1058642 (PMC9768184; doi:10.3389/fneur.2022.1058642)

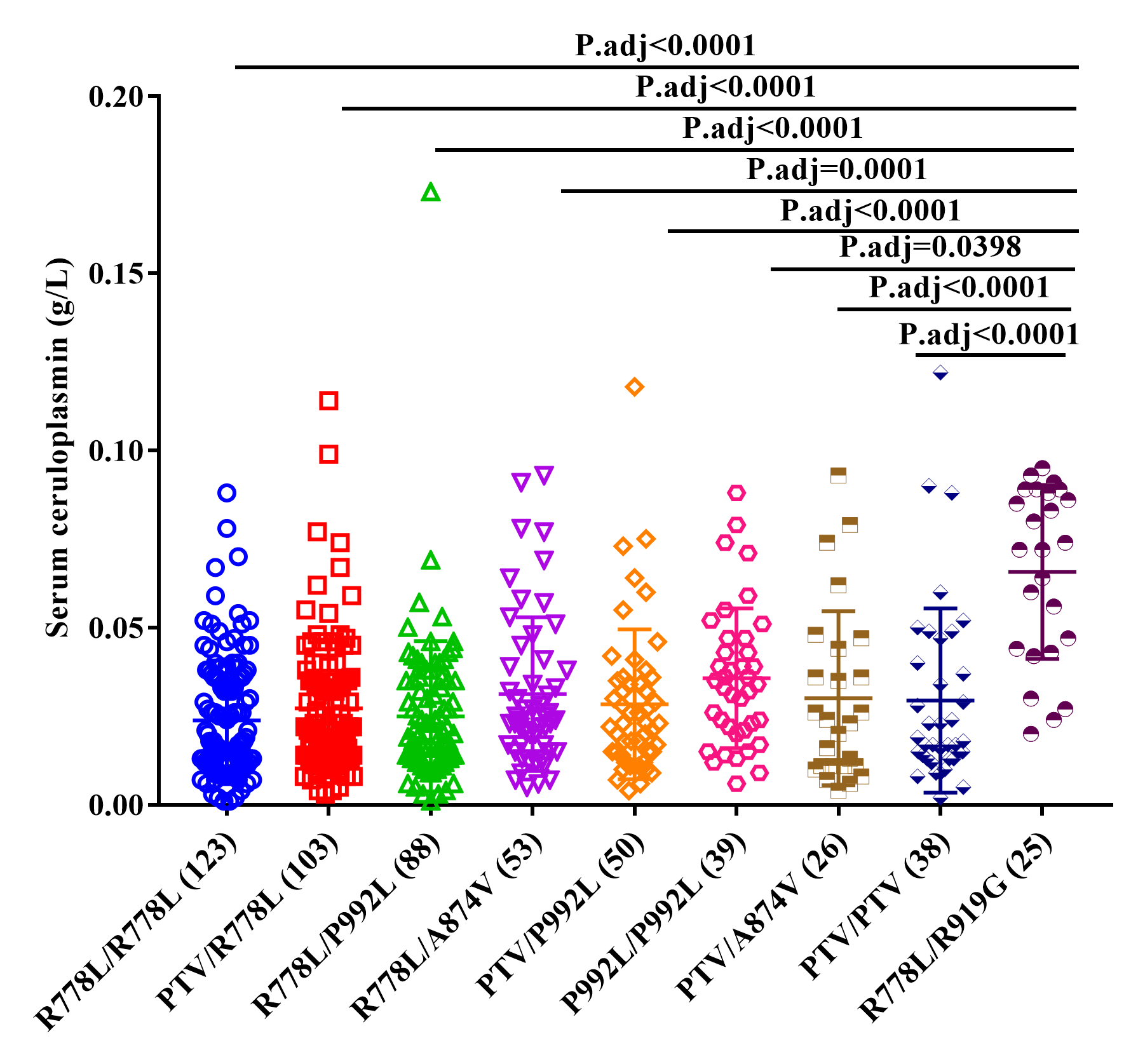

Supplement: Supplementary file 3 [file Image_1.tif]
